# Clinical Spectrum of *KCNA1* Mutations: New Insights into Episodic Ataxia and Epilepsy Comorbidity

**DOI:** 10.3390/ijms21082802

**Published:** 2020-04-17

**Authors:** Kelsey Paulhus, Lauren Ammerman, Edward Glasscock

**Affiliations:** Department of Biological Sciences, Southern Methodist University, Dallas, TX 75275, USA; kpaulhus@smu.edu (K.P.); lammerman@smu.edu (L.A.)

**Keywords:** episodic ataxia, epilepsy, *KCNA1*, Kv1.1

## Abstract

Mutations in the *KCNA1* gene, which encodes voltage-gated Kv1.1 potassium channel α-subunits, cause a variety of human diseases, complicating simple genotype–phenotype correlations in patients. *KCNA1* mutations are primarily associated with a rare neurological movement disorder known as episodic ataxia type 1 (EA1). However, some patients have EA1 in combination with epilepsy, whereas others have epilepsy alone. *KCNA1* mutations can also cause hypomagnesemia and paroxysmal dyskinesia in rare cases. Why *KCNA1* variants are associated with such phenotypic heterogeneity in patients is not yet understood. In this review, literature databases (PubMed) and public genetic archives (dbSNP and ClinVar) were mined for known pathogenic or likely pathogenic mutations in *KCNA1* to examine whether patterns exist between mutation type and disease manifestation. Analyses of the 47 deleterious *KCNA1* mutations that were identified revealed that epilepsy or seizure-related variants tend to cluster in the S1/S2 transmembrane domains and in the pore region of Kv1.1, whereas EA1-associated variants occur along the whole length of the protein. In addition, insights from animal models of *KCNA1* channelopathy were considered, as well as the possible influence of genetic modifiers on disease expressivity and severity. Elucidation of the complex relationship between *KCNA1* variants and disease will enable better diagnostic risk assessment and more personalized therapeutic strategies for *KCNA1* channelopathy.

## 1. Introduction

Mutations in the voltage-gated potassium channel gene *KCNA1* underlie a myriad of human diseases, thereby preventing simple genotype–phenotype correlations in patients. The primary disease associated with *KCNA1* mutations is episodic ataxia type 1 (EA1), a rare neurological movement disorder. Over half of the known *KCNA1* variants lead to EA1 only. Some patients with *KCNA1* variants exhibit EA1 in combination with epilepsy, while others suffer from epilepsy or epileptic encephalopathy in the absence of EA1. Finally, in some rare instances, *KCNA1* mutations cause hypomagnesemia, paroxysmal dyskinesia, and myokymia. To begin to decipher the complex genotype–phenotype relationships associated with *KCNA1* channelopathy and EA1, this review examines all known human *KCNA1* single nucleotide polymorphisms (SNPs) that have been identified as pathogenic or likely pathogenic to date. The disease phenotype associated with each variant is then examined to determine whether patterns exist between types of mutations and manifestation of disease. In addition, animal models of the *KCNA1* mutation are reviewed to provide further insight into phenotypic consequences of *KCNA1* dysfunction. Finally, the role of genetic modifiers in *KCNA1* channelopathy is discussed as another potential factor affecting genotype–phenotype relationships in patients.

## 2. Episodic Ataxia

Episodic ataxias (EAs) are a group of at least eight rare genetic neurological movement disorders (affecting approximately 1/100,000 people [1]). EAs belong to a larger family of paroxysmal movement disorders (PMDs), which are divided into two main types: ataxias and paroxysmal dyskinesias (PxDs) [2]. These two types of PMD are distinguishable by the nature of their attack. Whereas EAs are associated with ataxia characterized by impairments in purposeful movements (i.e., walking), speech (dysarthria), and vision (nystagmus), PxDs are associated with abnormal involuntary movement (dyskinesia), often involving postural abnormalities and repetitive movements (dystonia) [3,4]. EAs are categorized into eight main subtypes (EA1–8) based primarily on the underlying genetic cause. A ninth type of EA has recently emerged, termed “late onset” because it occurs around the fifth or sixth decade of life, but the cause of this form is still unknown [5]. The primary genes responsible for EA1, EA2, EA5, EA6, and EA8 have been identified as *KCNA1*, *CACNA1A*, *CACNB4*, *SLC1A3*, and *UBR4*, respectively [1]. Causative genes for the remaining types of EA (EA3, EA4, and EA7) have either been mapped to a chromosomal location or are entirely unknown. The forms of EA without associated genes are the most rare, so diagnosis relies on a unique combination of clinical characteristics to distinguish these forms from other EA subtypes [1].

Recurrent ataxic attacks are common to all EAs, but the characteristics of the disease vary significantly between the different EA subtypes and also between patients with the same causative gene mutation. For example, whereas patients with any EA type can exhibit vertigo during attacks, seizures are only reported in patients with EA1, EA2, EA5, and EA6 [1,6]. Both the age of onset and the attack duration are sources of variability between the EA types. Depending on the particular disorder, onset can occur anywhere from infancy through to late adult life, and attacks can last from seconds to days [1,5]. This same level of variability exists at the patient level, notably for patients sharing mutations in *KCNA1*, the genetic cause of EA1. Identical twins that share the same *KCNA1* gene mutation but exhibit different degrees of ataxic symptoms have been identified, suggesting that additional factors beyond the causative mutation may impact phenotype presentation [7]. Although EA1 is the most common diagnosis resulting from a *KCNA1* mutation, patients can also exhibit many other types of diseases such as epilepsy, hypomagnesemia, and paroxysmal kinesigenic dyskinesia (PKD) (Table 1). Why patients with mutations in the same gene exhibit such widely variable symptoms is not fully understood.

## 3. *KCNA1* Structure and Regulation

The *KCNA1* gene encodes the 495 amino acid (aa) Kv1.1 voltage-gated potassium (Kv) channel α-subunit [1,49]. *KCNA1* is one of 40 human Kv α-subunit genes that are spread across 12 different gene subfamilies (Kv1–12) [50]. Kv channels, such as Kv1.1, play important roles in regulating neuronal excitability by controlling the action potential shape, repolarization, and firing properties [51]. Kv1.1 is uniquely suited for counterbalancing depolarizing inputs and preventing excessive neuronal excitation because it has a much lower activation threshold and faster onset rate than other members of the Kv1 family, which includes Kv1.1–Kv1.8 [52]. Kv channels are comprised of four α-subunits which associate as homo- or hetero-tetramers to form a functional transmembrane pore [53,54,55]. To form a complete channel complex, α-subunit tetramers also associate with up to four accessory β-subunits that can impact channel gating, assembly, and trafficking [56]. In the brain, Kv1.1 associates with Kv1.2 and/or Kv1.4 α-subunits to form heterotetramers, but evidence is lacking for the presence of Kv1.1 homotetramers in the central nervous system (CNS) [57]. Although Kv1.1, Kv1.2, and Kv1.4 subunits are all abundant in the brain, their expression and channel composition varies depending on the brain region, cell type, and subcellular localization [58]. Interestingly, Kv1.1 levels are lowest in the cerebellum and hippocampus, implying a low copy number in heterotetramers in these brain regions [52]. The relatively low representation of Kv1.1 in these brain structures combined with its distinctive biophysical characteristics, which cannot be completely compensated for by other Kv1 subunits, has been hypothesized to cause a low functional reserve that renders the cerebellum and hippocampus especially vulnerable to Kv1.1 deficits [52].

Each Kv α-subunit has six transmembrane (TM) spanning segments (helices S1–S6) that include the functionally critical voltage-sensing and pore domains. Helices S1–S4 form the voltage-sensing domain of the protein, with S4 playing a specifically important role as the voltage sensor [59]. Evenly spaced positive charges across S4 allow this helix to acutely sense changes in voltage across the membrane; together with S3, these helices form a voltage sensor paddle that can change conformation to alter the state of the channel [59,60,61]. The pore region of the channel, which allows ion flux through the membrane, is formed by S5 and S6 [50]. The S4–S5 intracellular linker communicates changes in the voltage-sensing domain to the pore domain and thus can initiate a shift between the open and closed states of the pore [59,61]. The intracellular N- and C-terminal domains of each Kv α-subunit also regulate channel function. While the mechanisms are not fully understood, evidence suggests that the C-terminus influences channel tetramerization and membrane targeting [62], while the N-terminus participates in both channel inactivation and subunit assembly [63,64].

RNA editing of *KCNA1* transcripts is an important regulatory mechanism to control protein function. *KCNA1* transcripts can be edited by human adenosine deaminase acting on RNA 2 (ADAR2). ADAR2 converts adenosine to an inosine in the *KCNA1* transcript, thereby resulting in an isoleucine-to-valine substitution at amino acid (aa) 400 [65,66]. This editing process occurs normally in the brain, but the percentage of edited *KCNA1* transcripts varies by region. For example, in the mouse brain, approximately 20%, 35%, and 50% of transcripts are edited in the hippocampus, cortex, and cerebellum, respectively [66]. For this editing to take place, an RNA hairpin must form between complementary base pairs flanking the edit site on the transcript. ADAR2 then recognizes and enzymatically processes this hairpin as a substrate [65]. The aa at position 400, which is subject to RNA editing, is predicted to line the pore in S6 where it may interact with the inactivation particle of the channel contributed by β-subunits [66]. In support of this, functional biophysical studies have demonstrated that this RNA editing event decreases the channel recovery time from inactivation [65,66].

## 4. Overview of *KCNA1* Mutations in EA1 and Disease

**Figure 1:**
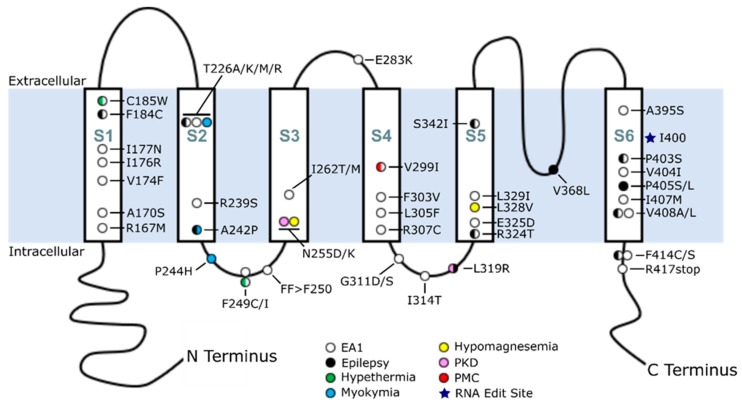
Map of *KCNA1* mutations associated with human disease. Human mutations in *KCNA1* were mapped across the protein and color-coded to indicate their clinically documented disease association. Circles with two colors represent mutations with multiple phenotypes. Multiple circles at a given amino acid position represent multiple diseases caused by different amino acid changes at the same position (e.g., N255D/K). Abbreviations: PMC, paradoxical myotonic congenita; PKD, paroxysmal kinesigenic dyskinesia.

EA1 was first identified in 1975 by Van Dyke and colleagues when they described a family with a movement disorder accompanied by myokymia, which is characterized by muscle rippling [14]; however, it was not until much later, in 1994, that Browne et al. identified *KCNA1* as the genetic cause of EA1 [11]. At present, the biomedical literature (Pubmed) and genetics databases (dbSNP and ClinVar) describe 47 *KCNA1* mutations identified in patients that are pathogenic or likely pathogenic (Table 1; Figure 1) [48]. These mutations span the Kv1.1 protein, from the first transmembrane region to the beginning of the C-terminal domain. The majority of these mutations are missense; however, one nonsense mutation (R417stop) and one in-frame deletion event (FF>F250) have also been identified [21,24]. Many of these mutations have been examined at the electrophysiological level to determine their functional consequences. These studies provide evidence that pathological *KCNA1* mutations lead to a loss-of-function (LOF) of Kv1.1 by various mechanisms [18,35]. Furthermore, some *KCNA1* mutations have a dominant negative effect whereby incorporation of the mutated α-subunit detrimentally impacts the otherwise normal subunits that form the tetrameric structure of the potassium channel [37,39]. In addition, biochemical and electrophysiological experiments have revealed that certain *KCNA1* mutations can lessen the surface expression of Kv1.1, reduce inactivation, or even decrease protein stability [67,68]. The varying functional consequences of these different *KCNA1* LOF mutations underscore the importance of understanding *KCNA1* at both the genetic and functional protein levels to understand how specific mutations may ultimately correlate with human disease phenotypes.

## 5. Phenotypic Variability Associated with *KCNA1* Mutations

With the discovery of a *KCNA1* mutation as the molecular cause of EA1, the gene became the first potassium channel gene associated with human disease [11]. Since that time, *KCNA1* mutations have also been associated with several other diseases, including epilepsy and hypomagnesemia. These diseases can occur alone or comorbid with EA1. The spectrum of phenotypes exhibited by patients with *KCNA1* mutations is broad, and some patients exhibit unique symptoms not shared by most others. Examining the different diseases caused by mutations in *KCNA1* and mapping the structural locations of these variants reveals patterns that may underlie the relationship between a specific genotype and phenotype.

### 5.1. EA1

The most well represented disorder associated with *KCNA1* mutations is EA1. At the cellular level, *KCNA1* is expressed in interneurons, including basket cells of the cerebellum. Basket cells form inhibitory synapses on Purkinje cells and provide the sole output from the cerebellar cortex [1,54]. In EA1, Kv1.1 dysfunction due to *KCNA1* mutation is thought to cause hyperexcitability of these interneurons, leading to excessive inhibition of Purkinje cells and subsequent motor deficits [1,49]. In EA1 patients, the age of onset is typically younger than 20 years, and attacks commonly consist of ataxia, myokymia, and dysarthria [1,16]. Attacks are usually triggered by stressors such as exercise, emotional stress, temperature, and sudden movement [16,31]. *KCNA1* mutations associated with EA1 occur widely across the protein, from the first transmembrane segment to the early C-terminal domain (Table 1; Figure 1). A subset of these mutations results in EA1, with unique comorbidities such as hyperthermia and seizures/epilepsy. Additional patient variability is apparent in cases that display myokymia/neuromyotonia in the absence of ataxia.

Hyperthermia, a somewhat rare feature in EA1, has been reported in two patients with different *KCNA1* mutations in the voltage-sensing domain: C185W and F249C [15,22]. Both mutations associated with hyperthermia lie in the voltage-sensing domain. C185 is located on the extracellular edge of S1, and F249 is located on the intracellular linker between S2 and S3. In contrast to hyperthermia associated with the F249C mutation, a F249I mutation causes EA1 without hyperthermia [11]. Thus, the specific amino acid change may be a contributing factor to differences in phenotype. It could also be attributable to environmental differences, as these mutations are found in separate families [11,22].

In unique cases, *KCNA1* mutations have been associated with myokymia or neuromyotonia without ataxia. These mutations are located on the S2 helix (T226K, A242P) and the S2–S3 intracellular linker (P244H) [8,18,21]. Interestingly, in the 16 amino acid span between these three mutations, there are two different mutations that cause a more common form of EA1 which presents with myokymia [11,19,21,23]. It is unclear why such closely localized mutations can lead to myokymia, neuromyotonia, or EA1 with myokymia. Mutations in the S2 transmembrane helix and the linker to the S3 transmembrane helix appear to be disproportionately represented in association with neuromyotonia or myokymia alone. Genetic modifiers or environmental factors may influence the patient phenotype, as there are four LOF mutations at amino acid position 226, but only one presents with myokymia; the other three mutations at this position exhibit EA1 or EA1 with epilepsy [12,17,18,19,20].

### 5.2. Epilepsy, Seizures, and Epileptic Encephalopathies

Epilepsy is over-represented in patients with EA1 and appears to be related to the impact of specific mutations on the function of the Kv1.1 pore region. Kv1.1 is widely expressed in neurons throughout the brain, including the hippocampus and cerebellum, as well as in the peripheral neuron system [54,69]. Subcellularly, it localizes to axons, including unmyelinated axons, axon initial segments, and juxtaparanodal regions of myelinated axons, where the protein plays an important role in action potential propagation, repetitive firing properties, and neurotransmitter release [51,54,69,70]. Neurons lacking functional Kv1.1 subunits exhibit membrane hyperexcitability at both subcellular (e.g., axons) and multicellular network levels (e.g., CA3 region of the hippocampus), which can manifest in the brain as epilepsy [71,72,73].

**Figure 2:**
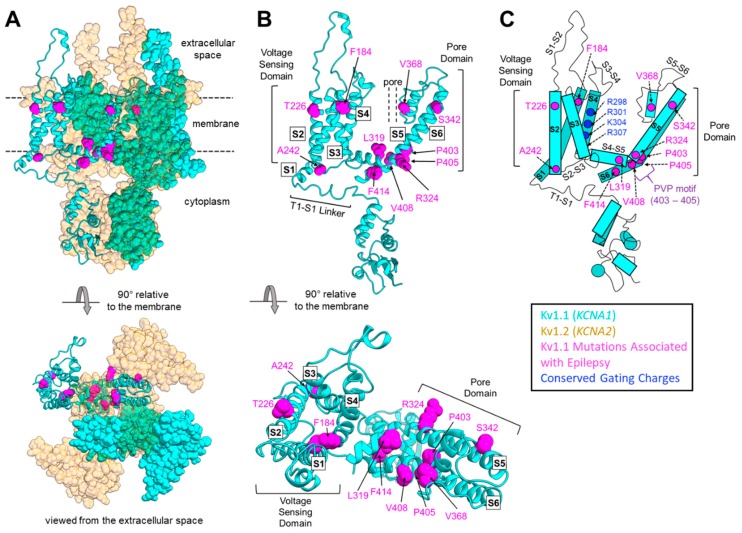
Modeling *KCNA1* mutations associated with epilepsy. (**A**) Model of a Kv1.1–Kv1.2 heterotetramer built using a homology model of human Kv1.1 (blue) and human Kv1.2 (orange), which were made with SWISS-MODEL [92] using the crystal structure of rat Kv1.2 (RCSB PDB: 3LUT) [74]. Human Kv1.1 and Kv1.2 share 80% and 99.4% sequence identity with rat Kv1.2, respectively. One Kv1.1 subunit is shown in ribbons, with epilepsy-associated mutations shown in van der Waals (vdW) representation colored magenta, and other subunits shown in vdW representation. (**B**) Homology model of a single Kv1.1 subunit with epilepsy-associated mutations shown in magenta in vdW representation. (**C**) Two-dimensional representation of a human Kv1.1 subunit. Magenta circles represent mutations associated with epilepsy; blue circles are conserved gating charges in the voltage-sensing domain. The highly conserved PVP motif is noted in purple.

In contrast with EA1-associated mutations, which span the whole length of Kv1.1, *KCNA1* mutations associated with epilepsy or a family record of seizures appear to preferentially localize in certain domains of the Kv1.1 protein, specifically in the S1/S2 helices and the pore domain (Table 1; Figure 1 and Figure 2). In patients with *KCNA1*-related epilepsy, three mutations have been identified in the S1 and S2 helices of the voltage-sensing domain. Several mutations have also been found throughout the pore domain, from the end of the S4–S5 intracellular linker to the beginning of the C-terminal domain (Table 1; Figure 2). In the S1 helix, an F184C mutation that impairs pore function has been found. F184 is a component of a “hydrophobic layer” formed by 10 conserved, buried residues in the voltage-sensing domain [74]. These hydrophobic residues interact with the conserved gating charges on the S4 helix and are thought to be the molecular basis for the creation of a focused electric field between internal and external solutions in the voltage-sensing domain [74]. F184 may also influence the selectivity filter in the adjacent subunit [14,75,76]. In the S2 helix, two different epilepsy/seizure-associated mutations (T226R and A242P) that are predicted to cause neuronal hyperexcitability have been identified [20,77]. Electrophysiological studies showed that the A242P variant reduces the K^+^ current amplitude, whereas T226R leads to an increase in neurotransmitter release [21,78]. These two residues (T226 and A242) surround a phenylalanine residue at position 232 (position 233 in Kv1.2) which stabilizes the pore’s open configuration [75,79,80,81]. Consequently, these two mutations may indirectly impact the ability of F232 to make the proper contacts required to stabilize the pore.

Epileptic encephalopathies (EEs) also arise from *KCNA1* mutations which directly impact pore function. EEs are defined by early onset seizures and epileptiform activity that progressively impairs brain function, leading to cognitive, behavioral, and language deficits [82]. Of special importance is the PVP (Pro-Val-Pro) motif of Kv channels; this motif (aa 403–405 in Kv1.1) is critical for flexibility in helix S6, as it allows proper gating of the channel [83]. A P403S mutation at the first proline of the PVP motif is associated with both EA1 and epilepsy with intellectual disability [43]. This mutation was identified in a set of identical twin boys with epilepsy, where one had a greater seizure burden and level of intellectual disability than the other [43]. The difference in expressivity between identical twins harboring the same mutation exemplifies the difficulty of determining the relationship between genotype and phenotype. With one having more severe symptoms than the other, it is possible that there could be an underlying de novo genetic modifier in one of the twins, causing the difference in phenotype. The recent advent of next generation sequencing is allowing the identification of small genetic differences responsible for phenotypic discordance between siblings with the same disease, thereby enabling molecular dissection of the effects of multilocus pathogenic variants on phenotypic variation [84]. Two different mutations in the second proline of the PVP motif in the S6 helix, P405S and P405L, have also been identified in EE patients [43]. De novo mutations in the homologous position in *KCNA2* also cause EE in patients [85]. Interestingly, mutation of the valine in the PVP motif (V404I) is associated with EA1 without epilepsy and only mild intellectual disability, suggesting alteration of the prolines may cause higher risk for the development of epileptic encephalopathy or epilepsy with intellectual disability [44]. The only other clinical case of *KCNA1* epileptic encephalopathy was caused by a V368L mutation [42]. While not in the PVP motif, this mutation sits behind the selectivity filter of the pore, helping to support its structure [86]. Of note, a T374A mutation in this same region in *KCNA2* also causes EE [87].

The importance of the PVP motif in epilepsy is further strengthened by high resolution genomic screening, which has also identified a *KCNA1* copy number variant (CNV) affecting this region in a three year old child with severe myoclonic epilepsy of infancy (SMEI) who succumbed to sudden unexpected death in epilepsy (SUDEP) [88]. The patient exhibited drug-resistant EE with noted cardiorespiratory complications and global developmental delay [88]. Post mortem analysis showed that the patient harbored non-synonymous SNPs and CNVs in several different ion channel genes, including a de novo non-synonymous SNP (A1783V) in the voltage-gated sodium channel *SCN1A* which was previously found in another SMEI patient [88].The CNV in *KCNA1* resulted in five extra copies of the region that extends from the PVP motif to the end of the S6 transmembrane helix, which may render the protein greatly impaired or non-functional [88]. This region of the S6 helix connects to the flexible C-terminal domain, which may influence channel tetramerization and membrane targeting [62]. The addition of a long flexible piece so close to the C-terminal domain could hinder channel tetramerization and critical interactions with other subunits, or it could prevent proper protein folding and embedding into the plasma membrane. Furthermore, motions of this flexible addition could disrupt the conformation, tilt, and orientation of the S6 helix, and thus, adversely affect the pore domain of the channel. Thus, the *KCNA1* CNV is likely a key contributor to both the EE and SUDEP phenotypes, especially given the strong association between missense mutations in the PVP motif and severe forms of epilepsy and intellectual disability. However, the principal risk factor for epilepsy and premature death in the patient was probably the co-occurrence of the *KCNA1* mutation with the non-synonymous SNP in *SCN1A* [88]. Mutations in *SCN1A* are the most common genetic cause of SMEI, and they are strongly implicated in EE and SUDEP [89,90]. Therefore, the combination of variants in *KCNA1* and *SCN1A* could have led to a lethal compound effect.

Several epilepsy and seizure-associated mutations (P403S, P405S, P405L, and V408L) are caused by underlying nucleotide changes in the region of the *KCNA1* mRNA transcript that is edited by ADAR2 (Table 1). These sequence changes may disrupt RNA base pairing within or near the region that forms a hairpin. By preventing proper formation of the hairpin in the *KCNA1* transcript, these mutations may prevent subsequent RNA editing by ADAR2 and thereby impair an important regulatory mechanism for Kv1.1 protein function [66]. As mentioned previously, residues P403 and P405 are structurally critical in the S6 PVP motif, so the combined effects of these mutations (P403S and P405S/L) on both RNA editing and subsequent channel function may explain the more severe phenotypes seen in these patients.

Unlike the other mutations in the RNA-editing region of the transcript, the V404I, I407M, and V408A variants do not cause seizures (Table 1). Mutational experiments show that the nucleotide changes underlying both epilepsy-associated (V408L) and non-epilepsy-associated (I407M and V408A) variants reduce editing of the *KCNA1* mRNA transcript by disrupting the complementarity required for hairpin duplex formation [65]. The same studies showed that mutations affecting nucleotides in the region immediately flanking the RNA editing site, while not predicted to alter the secondary structure of the hairpin, still diminish editing by ADAR2 [65]. Thus, the efficiency of editing by ADAR2 is determined by more than simply the structure of the hairpin. In a rat model, RNA editing reduces the ability of 4-aminopyridine (4-AP), a Kv1-channel blocker, to induce seizures [91]. This further suggests that RNA editing of *KCNA1* plays a protective role against seizures and that loss of RNA editing could increase seizure susceptibility.

In summary, a total of nine mutations in the S5–S6 pore domain and three mutations in the voltage-sensing domain are associated with epilepsy or seizures (Figure 2) [7,37,41,46]. Table 1 and Figure 2 show that these epileptic/seizure phenotypes are more prevalent for mutations in the pore domain of the channel, suggesting that epilepsy is more likely to manifest with *KCNA1* mutations that affect pore function, either directly or indirectly. The mutations in the more peripheral segments of the protein that also produce epileptic phenotypes fit with this hypothesis, as they could directly (for mutations in S1) or indirectly (for mutations in S2) disrupt important residues known to interact directly with the selectivity filter or pore or with other subunits of the tetrameric channel [75,76,79,80,81]. Mutations in the PVP motif of the pore domain can directly disrupt pore structure and function, whereas mutations that alter RNA base pairing, which may prevent proper hairpin formation and RNA editing of the channel transcript, can indirectly affect pore function [43,65,66].

### 5.3. Hypomagnesemia

Hypomagnesemia is not well represented among disorders caused by *KCNA1* mutations, which complicates subsequent analysis of genotype–phenotype relationships. Hypomagnesemia is characterized by decreases in serum magnesium leading to symptoms of tremor, tetany, and muscle weakness [26,39]. The kidneys play a critical role in the reabsorption of magnesium, with the distal convoluted tubule (DCT) acting as the final reabsorption center and determining the concentration of magnesium that is ultimately excreted from the body [93]. The transient receptor potential cation channel, subfamily M, member 6 (TRPM6) is critical to magnesium reabsorption in the DCT and utilizes an electrochemical gradient to drive magnesium flow [93,94]. Kv1.1 is expressed alongside the magnesium transporter TRPM6 in the distal convoluted tubules of the kidney, where it mediates K^+^ flow to regulate the electrochemical gradient [25,26]. Two autosomal dominant *KCNA1* mutations, N255D and L328V, have been linked to hypomagnesemia. These are located in the voltage-sensing domain on the S3 helix and in the pore domain on the S5 helix, respectively [26,39]. Since only two *KCNA1* mutations causing hypomagnesemia have been identified, the relationship between mutation and phenotypic presentation is unclear. One possibility is that the incidence of hypomagnesemia in patients with *KCNA1* mutations is greater than reported due to ascertainment bias from patients not being clinically examined for this disorder.

### 5.4. Paroxysmal Kinesigenic Dyskinesia

Two *KCNA1* mutations have been associated with paroxysmal kinesigenic dyskinesia (PKD), another type of paroxysmal movement disorder along with EA. Interestingly, these two mutations, N255K and L319R, are in different domains of the Kv1.1 protein [27]. N255 is located on the S3 helix; L319 sits between S4 and S5 in an intracellular linker that connects the voltage-sensing domain to the channel pore [95]. The L319R patient also exhibited seizures, providing evidence for the hypothesis that pore-related mutations are likely to be comorbid with epilepsy. [27]. With a limited number of cases, it is difficult to speculate on the relationship between these mutations and the PKD phenotype, but it does increase the phenotypic variability of patients with *KCNA1* mutations, which may include different paroxysmal movement disorders.

## 6. Understanding *KCNA1* Mutations Using Animal Models

### 6.1. V408A/+ Mouse Model

Beyond the analysis of human cases, animal models can also further our understanding of the effects of specific *KCNA1* mutations (Table 2). The most relevant animal model of human EA1 is found in mice engineered to have a V408A mutation, a homolog of the original V408A human mutation found to cause EA1 [11,96]. When mice are homozygous for the mutation, they die embryonically between days E3 and E9 [96]. However, when *Kcna1* is globally knocked out, mice live past the embryo stage and do not begin dying until around the third week of life [73]. This suggests that the V408A mutation has a dominant negative effect that is more deleterious than the complete absence of the gene. The V408A/+ mice exhibit stress-induced motor incoordination similar to clinical cases with the same mutation [96,97]. Therefore, the same mutation.

### 6.2. Megencephaly Mouse Model

The *megencephaly* (*mceph/mceph*) mouse model is a *Kcna1* mutant with an 11 bp deletion, resulting in a truncation at amino acid 230, which leads to the production of a protein that only includes the N-terminus, S1, and first extracellular loop [98,99,100]. The *mceph/mceph* mice display megalencephaly with an enlarged brain specifically in the hippocampal and cortical regions due, in part, to increased numbers of glia and neurons in these regions [98,100,108]. The *mceph/mceph* mice also exhibit seizures, suggesting that the increase in cell number may either predispose them to epilepsy or be a consequence of the seizures [98]. While not modeled after a specific human case, the *mceph/mceph* mice are phenotypically similar to a patient with a P405S mutation who exhibited mild macrocephaly and seizures as part of an EE [43,98]. Thus, more severe *Kcna1* mutations, such as the large truncation in *mceph/mceph* mice, may share phenotypic similarities with missense mutations in the critical pore region.

### 6.3. Kcna1 Global Knockout Mouse Model

Another model, the *Kcna1*-null mouse, has provided substantial insight into the importance of Kv1.1 in epilepsy, sleep, and cardiorespiratory function. Rather than a missense mutation or truncation, these mice lack *Kcna1* globally due to targeted gene deletion [72]. Homozygous knockout mice are viable at birth, but begin dying between two to three weeks of age, with only a small proportion (~25%) surviving to ten weeks depending on their genetic background [72,73]. Corresponding with the timing of early lethality in these animals is the onset of spontaneous limbic seizures which contribute to death by impairing cardiorespiratory function; thus, these mutants are useful model for the study of SUDEP [72,73,101,109].

While not representative of a specific clinical mutation, this model recapitulates the sleep and respiratory phenotypes reported in some EA1 patients. For example, a patient with a T226R mutation in *Kcna1* presented with reduced sleep efficiency and episodes of obstructive sleep apnea [19]. In addition, another patient with a C185W mutation exhibited a short sleep phenotype, needing only five or six hours of sleep while being very active during the night [15]. Finally, an EA1 family member with the FF>F250 mutation exhibited attacks in which they were unable to catch their breath [23]. *Kcna1*-null mice exhibit sleep deficits, including more fragmented sleep and reductions in the time spent in rapid eye movement (REM) and non-rapid eye movement (NREM) sleep [101,110,111]. *Kcna1*-null mice also display abnormal respiration during non-seizure periods, including increases in apnea frequency, breathing rate, and respiratory variability and reductions in sigh–apnea coupling and oxygen saturation levels [101,109]. Thus, *Kcna1* appears to be important for the normal regulation of sleep and respiration, suggesting that EA1 patients or others with known *KCNA1* mutations should be evaluated for deficits in these areas.

Lastly, *Kcna1*-null mice show numerous cardiac abnormalities which may reflect both heart-extrinsic and heart-intrinsic mechanisms. During non-seizure periods, *Kcna1*-null mice exhibit increases in the frequency of atrioventricular (AV) conduction blocks and heart rate variability, a measure of the autonomic control of the heart [102]. Vagal mechanisms likely contribute to these abnormalities, since Kv1.1-deficient vagus nerves exhibit hyperexcitability, and conditional knockout mice lacking Kv1.1 in neurons (see discussion below) also display augmented heart rate variability [102,106]. The expression of Kv1.1 channels in cardiomyocytes in both mice and humans suggests that the channels can also influence cardiac function directly [103,112]. Electrophysiological studies have confirmed a functional role for Kv1.1 channels in the heart where they contribute to outward K^+^ currents and action potential repolarization in atrial cardiomyocytes [103]. Furthermore, the absence of Kv1.1 channels in mice causes susceptibility to inducible atrial fibrillation [103]. Traditionally, Kv1.1 channels have been considered primarily neuronal and absent in the heart [112], which may explain the low number of EA1 cases reported with a detailed cardiac examination. The *Kcna1*-null mouse model shows that Kv1.1 subunits are influential in many vital processes in the body and should be better assessed in clinical cases involving *KCNA1* mutations to identify patients at risk for severe complications.

Interestingly, *Kcna2*-null mice, which lack Kv1.2 subunits, also exhibit epilepsy and premature death phenotypes, but they appear more severely than in *Kcna1*-null mice. *Kcna2*-null mice begin exhibiting spontaneous seizures by about postnatal day 15, similar to the age of seizure onset in *Kcna1*-null mice [113]. Seizures in *Kcna2*-null mice exhibit characteristics of brainstem involvement, including a sudden explosive onset of wild running and jumping, followed by tonic extension (TE) that lasts several seconds and leads to death in 50% of animals, probably due to respiratory arrest associated with TE [113]. In contrast, the seizures in *Kcna1*-null mice exhibit signs of limbic origin similar to the behaviors of the Racine scale, including a progression from immobility, facial twitching, and head nodding to forelimb clonus followed by rearing and falling and sometimes wild running and jumping [114]. Although seizures begin at similar ages in *Kcna1*-null and *Kcna2*-null mice, lifespans are much shorter in *Kcna2*-null animals with all dying by 19 days old [113]. In addition, prior to death, juvenile *Kcna2*-null mice spend significantly less time in NREM sleep and significantly more time awake, similar to observations in *Kcna1*-null animals [101,110,111,115]. Thus, in mice, the absence of Kv1.2 subunits appears to cause a more severe seizure phenotype than Kv1.1 deficiency, possibly due to differences in the function, temporal expression, or localization of the subunits, as well as the ability of other subunits to adequately compensate [58].

### 6.4. Kcna1 Neuron-Specific Conditional Knockout Mouse Model

Recently, neuron-specific *Kcna1* conditional knockout (cKO) mice were developed to more precisely understand the role of *Kcna1* in epilepsy and sudden death. To generate these mice, floxed *Kcna1* mice were crossed with Synapsin1-Cre mice to specifically delete *Kcna1* in the neurons of the brain [106]. These mice recapitulated the global knockout animals, suggesting that neuronal *Kcna1* is largely responsible for these phenotypes [106]. It should be noted, however, that the phenotypes were not as severe as those of the global knockout mice, suggesting that, while neuronal Kv1.1 is critical for these phenotypes, Kv1.1 expression in other cells, such as cardiomyocytes and glia, may also make a functional contribution [106]. As *Kcna1* dysfunction is known to cause diseases of the brain (epilepsy, ataxia) and kidney (hypomagnesemia), it makes sense that Kv1.1 subunits in other regions of the body play individually critical roles. Future studies utilizing different conditional knockout strains will aid in the understanding of the tissue-specific contributions of *Kcna1* to disease.

### 6.5. S309T Rat Model

A rat *Kcna1* mutation that recapitulates human phenotypes, despite not being modeled after a clinical case, has been identified. In this model, *N*-ethyl-*N*-nitrosourea (ENU) mutagenesis generated a missense mutation, S309T, in the S4 voltage sensor segment of *Kcna1* [107]. Rats carrying this S309T mutation exhibit myokymia, neuromyotonia, and spontaneous epileptic seizures [107]. Thus far, it is the only rodent model that has been shown to recapitulate electromyography (EMG)-detectable myokymia [107]. While there are no human mutations at this specific position, there are some in close proximity, such as L305F, which is also associated with ataxia and neuromyotonia [34,107]. As seen in Table 1 and Table 2, in humans, the mutations surrounding this position do not exhibit seizure phenotypes, as the rat model does. This could be as a result of genetic modifiers or strain differences specific to the rat, but without a clinical case harboring the same mutation it is difficult to speculate with certainty.

## 7. Genetic Modifiers Add to Genotype–Phenotype Complexity

In addition to the exact location and nature of mutations, another factor that could influence *KCNA1* genotype–phenotype correlations is the presence of genetic modifier mutations that could exacerbate or suppress disease penetrance or expressivity. In support of this, several gene mutations that modify epilepsy phenotypes in *Kcna1*-null mice have been identified. The first genetic modifier identified was the mouse *tottering* (*tg*) mutation, a partial LOF allele of the P/Q-type calcium channel α-subunit *Cacna1a*, which was crossed into *Kcna1*-null mice. The *tg* mice display spike-wave absence-like seizures as well as ataxia [116,117]. Double mutant *Kcna1*^−/−^; *Cacna1a^tg/tg^* mice exhibit reduced seizure frequencies and drastically improved survival [73]. Thus, a partial LOF mutation in a gene implicated in EA2 (i.e., *Cacna1a*) alleviates abnormalities due to the absence of the gene responsible for EA1 (i.e., *Kcna1*). Another study identified *Mapt* as a modifier of *Kcna1* [118]. *Mapt* encodes the microtubule binding protein tau, which is known to regulate microtubule stability and axonal trafficking and is implicated in Alzheimer’s disease [119,120]. *Kcna1*^−/−^ mice carrying *Mapt^−/−^* mutations exhibit reductions in seizures and improved survival [118]. The BCL2-associated agonist of cell death (*Bad*) gene has also been identified as a third genetic modifier of *Kcna1* [121]. *Kcna1*^−/−^; *Bad*^−/−^ mice outlive *Kcna1*^−/−^ mice and exhibit reduced seizure severity [121]. Finally, heterozygosity for a deletion in the *Scn2a* voltage-gated sodium channel α-subunit gene acts as a protective modifier in *Kcna1*-null mice, decreasing the seizure burden while improving lifespan and brain–heart dynamics [122]. These genetic interactions demonstrate that *Kcna1*-related phenotypes can be dramatically modified by second-site gene mutations.

Further evidence of potential genetic modifiers can be observed by the presence of phenotypic differences between *Kcna1*-null mice maintained on different genetic backgrounds. While all *Kcna1*-null mice exhibit epilepsy and sleep deficits, they can also manifest differences in mortality and respiratory deficits depending on the genetic background of the mouse strain. *Kcna1*-null mice with a C3HeB/FeJ genetic background begin seizing and dying at about 5 to 7 weeks old, leading to a mortality rate of 100% [104,105]. In contrast, *Kcna1*-null mice with a Black Swiss genetic background exhibit seizures and death with an earlier onset of 2 to 3 weeks old, but only about 75% of animals die prematurely, usually by the 6th week of life [72,73]. The two strains also exhibit potential differences in respiration. C3HeB/FeJ-strain knockout mice display increases in apnea frequency, whereas Black Swiss-strain knockout mice show a decrease [101,109]. These examples of strain variation provide additional evidence that genetic modifiers can alter the penetrance and expressivity of phenotypes associated with *Kcna1* mutations. However, environmental factors may also influence phenotypic differences between *Kcna1*-null strains since the mice are maintained in different laboratory facilities.

## 8. Non-Coding and Benign *KCNA1* Mutations

The complete list of recorded *KCNA1* variants is much more extensive than the 47 mutations given in Table 1. The full list of known *KCNA1* variants in ClinVar contains >100 additional mutations in the gene’s untranslated regions (UTRs) which are categorized as benign, likely benign, or of uncertain significance. Additionally, more than 30 mutations, mostly synonymous single nucleotide polymorphisms, have been identified in the N- and C-terminal regions of the protein which are also classified as benign, likely benign, or of uncertain significance. All pathogenic or likely pathogenic mutations in *KCNA1* localize between the beginning of the first TM region and the early C-terminal domain. Thus, perhaps unsurprisingly, the likelihood of a mutation being pathogenic is greater when it occurs in the transmembrane regions of the protein and the flexible linkers that join them. However, it should be noted that in the transmembrane regions of the protein and their adjoining linkers, >10 mutations that are classified as non-pathogenic or as unlikely to be pathogenic have been found. Five of these mutations have been designated as benign or likely benign since they result in silent mutations where the amino acid remains unchanged. Two others are listed as being of uncertain significance even though they disrupt critical residues at positions 403 and 408, which are also associated with known pathogenic mutations. Importantly, the *KCNA1* nucleotide sequences that encode these aa residues at positions 403 and 408 surround the important RNA editing region of the *KCNA1* mRNA transcript, which makes it tempting to speculate they could be pathogenic by increasing seizure susceptibility. Furthermore, aa 403 is also part of the highly conserved PVP motif, where a mutation results in EE [43]. More clinical evaluation of patients harboring these mutations of uncertain significance would help to clarify the relationship between mutation location and phenotypic consequences.

## 9. Unanswered Questions and Conclusions

This review sheds light on a potential location bias for *KCNA1* mutations underlying epilepsy which could help to explain why seizures are a common comorbidity in EA1 patients; however, several questions still need to be explored. Why are all *KCNA1* variants associated with epilepsy limited to the S1 and S2 helices in the voltage-sensing domain and to the pore domain? Perhaps certain mutations, especially those in the voltage-sensing S4 helix where many highly conserved residues lie, are lethal and thus not seen in patients. Although all pathogenic *KCNA1* mutations identified result in channel LOF, do the epilepsy-associated mutations impair Kv1.1 function more severely? Electrophysiological studies suggest that the epilepsy-causing mutations cannot always be predicted by simply assessing which mutations have the most deleterious effects on channel function. For example, four mutations have been identified at amino acid position 226 on the S2 helix (T226A/K/M/R), and only T226R presents with epilepsy [12,17,18,19,20]. Both the epilepsy-causing T226R mutation are non-functional and act in a dominant negative fashion, yet they lead to different phenotypes [18,20]. Thus, the location, specific amino acid change, and consequence of a mutation on channel function likely all play interconnected roles in determining the phenotypic presentation of the mutation. What are the relative contributions of genetic modifiers and environmental factors to the phenotypic heterogeneity seen with *KCNA1* channelopathy? What types of genes can modify *KCNA1*-related disease, and can they be exploited as therapeutic targets for disease treatment? Since Kv1.1 subunits have only recently been identified as playing a role in regulating cardiac electrophysiology, will new patients be identified that exhibit cardiac dysfunction associated with *KCNA1* mutations? The entirety of the cardiorespiratory system should be evaluated closely in patients with *KCNA1* channelopathy, especially in cases involving epilepsy, as seizures in *Kcna1*-null mice evoke respiratory abnormalities that appear to drive cardiovascular dysfunction, potentially increasing the risk of SUDEP [101,102]. Given the great phenotypic variability associated with *KCNA1* channelopathy, how many disease-causing variants, both SNPs and CNVs, have been missed or overlooked in gene sequencing studies as a result of sampling bias? Elucidation of answers to these questions and others will require a multi-pronged approach, including the generation of new animal models of clinically-relevant mutations, in-depth electrophysiological and mutagenesis studies to identify critical regions of Kv1.1, and a larger database collection of *KCNA1* mutations in patients.

In summary, *KCNA1* channelopathy exhibits broad variability in clinical disease presentation that appears to be influenced by the location and nature of the mutated amino acid residue. Epilepsy and seizure phenotypes appear to be restricted to mutations affecting the S1/S2 helices and pore domain of the Kv1.1 subunit, providing a possible explanation for epilepsy comorbidity in a subset of EA1 patients. Animal models demonstrate that genetic modifiers of *KCNA1* mutations can also significantly impact disease manifestation and severity. Moving forward, improved understanding of *KCNA1* genotype–phenotype relationships at the molecular and electrophysiological levels in animal models and patients will enable more precise clinical diagnostic assessment and better therapeutic strategies for *KCNA1* channelopathy.

## Figures and Tables

**Table 1:** Human *KCNA1* mutations designated as “pathogenic” or “likely pathogenic” with heterogenous phenotypes.

Mutation	Protein Domain	Clinical Diagnoses	Other Clinical Observations	Reference
R167M	S1	EA1		[8]
A170S	S1	EA1		[9]
V174F	S1	EA1		[10,11]
I176R	S1	EA1		[12]
I177N	S1	EA1		[13]
F184C	S1	EA1 + Seizures		[14]
C185W	S1	EA1 + Hyperthermia	Sleep ^a^	[8,15,16]
T226A	S2	EA1		[12]
T226M	S2	EA1		[17]
T226K	S2	Myokymia		[18]
T226R	S2	EA1 + Epilepsy	Respiratory ^b^, Sleep ^c^, DD	[19,20]
R239S	S2	EA1		[11]
A242P	S2	Neuromyotonia + Seizures		[8,21]
P244H	S2–S3 IL	Myokymia		[21]
F249C	S2–S3 IL	EA1 + Hyperthermia		[22]
F249I	S2–S3 IL	EA1		[11]
FF>F250	S2–S3 IL	EA1	Respiratory ^d^	[23,24]
N255D	S3	Hypomagnesemia		[25,26]
N255K	S3	PKD		[27]
I262T	S3	EA1		[28,29]
I262M	S3	EA1		[30]
E283K	S3–S4 EL	EA1		[31]
V299I	S4	EA1 + PMC		[32]
F303V	S4	EA1		[33]
L305F	S4	EA1		[34]
R307C	S4	EA1		[7]
G311D	S4–S5 IL	EA1		[35]
G311S	S4–S5 IL	EA1		[36]
I314T	S4–S5 IL	EA1		[19]
L319R	S4–S5 IL	PKD + Seizures		[27]
R324T	S5	EA1 + Epilepsy		[37]
E325D	S5	EA1		[38]
L328V	S5	Hypomagnesemia		[39]
L329I	S5	EA1		[40]
S342I	S5	EA1 + Seizures		[28,41]
V368L	S5–S6 pore loop	EE	Severe ID	[42]
A395S	S6	EA1		*
P403S	S6 (PVP)	EA1 + Epilepsy	Respiratory ^e^, DD, Moderate ID	[43]
V404I	S6 (PVP)	EA1	Mild ID	[12,21,44]
P405S	S6 (PVP)	EE	DD, Macrocephaly ^f^	[43]
P405L	S6 (PVP)	EE	PDD ^g^	[43,45]
I407M	S6	EA1		[8]
V408A	S6	EA1		[11]
V408L	S6	EA1 + Seizures	Global DD	[46]
F414C	C Terminus	EA1		[47]
F414S	C Terminus	EA1 + Epilepsy		[16]
R417stop	C Terminus	EA1		[21]

Human SNP mutations were identified using the NCBI ClinVar and dbSNP databases. The full list of *KCNA1* mutations was filtered by the categories “Pathogenic” and “Likely Pathogenic.” The compiled list of human mutations was used as search criteria in PubMed to find clinical discussions of patients with these mutations and the functional research associated with them. Additional literature searches were also used to identify mutations not yet listed in the NCBI genetic databases. A compilation of “Benign”, “Likely Benign”, and “Uncertain Significance” mutations was also accomplished through the NCBI database ClinVar [48]. Abbreviations: IL, intracellular linker; EL, extracellular linker; PVP, proline-valine-proline motif; PKD, paroxysmal kinesigenic dyskinesia; EE, epileptic encephalopathy; PMC, paradoxical myotonic congenita; DD, developmental delay; ID, intellectual disability; PDD, pervasive developmental disorder. * published citation could not be found; ClinVar variation label NM_000217.3(*KCNA1*):c.1183G>T (p.Ala395Ser) and accession number VCV000431378.; ^a^ self-reported needing only 5–6 h of sleep per night and being very active during the night; ^b^ recurrent apneic episodes with cyanosis; ^c^ prolonged sleep latency, reduced sleep efficiency, obstructive sleep apnea, hypopnea, ~80% oxygen desaturation during sleep; ^d^ difficulty breathing during attacks and isolated episodes of an inability to inhale; ^e^ before age 2, very loud breathing at night; ^f^ head circumference in the 93rd percentile; ^g^ now also called autism spectrum disorder.

**Table 2:** Rodent models of *Kcna1* mutations and their associated phenotypes.

Species	Mutation	Phenotype	Reference
Mouse	V408A/+	Stress-induced motor incoordination, acetazolamide responsive	[96]
Mouse	Truncation at aa 230	Epilepsy, megalencephaly, unsteady gait	[98,99,100]
Mouse	Global gene knockout	Epilepsy, sleep deficits, cardiorespiratory abnormalities, sudden death	[72,73,101,102,103,104,105]
Mouse	Neuron-specific conditional gene knockout	Epilepsy, cardiorespiratory abnormalities, sudden death	[106]
Rat	S309T	Myokymia, neuromyotonia, seizures, premature death	[107]

Animal models of *Kcna1* dysfunction were identified through PubMed literature searches. Information was pooled from multiple sources, when applicable, to generate the summarized data presented in the table.

## Abbreviations

EA1	Episodic Ataxia Type 1
SNP	Single nucleotide polymorphism
TM	Transmembrane
CNS	Central nervous system
aa	Amino acid
vdW	van der Waals
PVP	Proline-Valine-Proline motif
IL	Intracellular Linker
EL	Extracellular Linker
PKD	Paroxysmal Kinesigenic Dyskinesia
EE	Epileptic Encephalopathy
PMC	Paradoxical Myotonic Congenita
DD	Developmental Delay
ID	Intellectual Disability
PDD	Pervasive Developmental Disorder
CNV	Copy number variant
SMEI	Severe myoclonic epilepsy of infancy
TE	Tonic extension
